# Role of depleted initial energy reserves in early benthic phase mortality of six marine invertebrate species

**DOI:** 10.1002/ece3.7723

**Published:** 2021-06-06

**Authors:** Shannon R. Mendt, Louis A. Gosselin

**Affiliations:** ^1^ Department of Biological Sciences Thompson Rivers University Kamloops BC Canada

**Keywords:** early benthic phase, energy reserves, juvenile benthic invertebrates, mortality factors, recovery, recruitment, starvation, survivorship, tolerance thresholds

## Abstract

Insufficient energy reserves are widely considered to be a primary factor contributing to high rates of early benthic phase mortality among benthic marine invertebrates, but this hypothesis has been based mostly on indirect, observational evidence, and remains largely untested. We therefore examined the role of initial energy reserves in regulating survivorship and growth during the early benthic phase. Recently settled or hatched individuals of six invertebrate species were collected from natural populations, maintained without food, and their survivorship was monitored. Contrary to expectations, starved individuals of all six species had high survivorship through the critical first 10 days of the early benthic phase, with half of the species experiencing <2% mortality, and the remaining three species experiencing only 6%–12% mortality. For five of the six species, 50% mortality was reached only after ≥50 days of starvation. Additionally, no difference in short‐term survivorship was detected among starved individuals of three different size classes (a proxy for energy reserves) of *N*. ostrina hatchlings. Finally, the effect of different durations of delayed feeding (0–50 days) on recovery (i.e., growth and survivorship) once food was made available revealed that duration of starvation prior to feeding can nevertheless have significant longer‐term impacts on the proportion of individuals that survive or their ability to grow. Together, these findings suggest that depleted energy reserves are not a primary cause of high mortality at the start of the early benthic phase, as had previously been hypothesized. Levels of energy reserves did influence growth, however, suggesting a possible indirect influence on performance by leaving individuals vulnerable for longer periods.

## Graphical abstract

We examined the role of initial energy reserves in regulating survivorship and growth during the early benthic phase (EBP) in six invertebrate species. Contrary to expectations, starved individuals of all six species had high survivorship through the first 10 days of the EBP, but starvation did affect the ability to grow when food became available. Thus, depleted energy reserves would not be a significant cause of EBP mortality, nor would individuals require immediate access to food to survive the critical first of early benthic life.

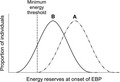

## 1 INTRODUCTION

Recruitment rates of benthic marine invertebrates are highly variable and can heavily impact the abundance and distribution of adult populations (Connell, 1985; Hunt & Scheibling, 1998; Miller & Waldbusser, 2016; Stoner, 1990). This is largely due to high rates of mortality during the first few days of independent benthic life (Ellien et al., 2000; Gosselin & Qian, 1997; Guillou & Tartu, 1994; Hadfield, 1986; Hunt & Scheibling, 1997; Keough & Downes, 1982; Osman et al., 1989; Thorson, 1966), a period identified as the early benthic phase (EBP) that encompasses early juvenile life and may also include premetamorphic larval life (Sandee et al., 2016). In addition, EBP mortality rates are highly variable not only among species, but also among and within cohorts of the same species (Gosselin & Qian, 1996; Jarrett, 2000; Jarrett & Pechenik, 1997; Phillips, 2017). The mechanisms responsible for the high rates and extensive variability in EBP mortality are not yet fully understood and remain an area of active research.

A factor that has been proposed as a major cause of mortality during early benthic life is depleted energy reserves, when individuals begin the EBP with minimal energy stores (Gosselin & Qian, 1997; Hunt & Scheibling, 1997; Jarrett & Pechenik, 1997). Energy reserves at the start of independent benthic life are known to affect other relevant aspects of EBP performance, including the subsequent size and growth rate of individuals throughout juvenile life (Emlet & Sadro, 2006; Miller & Emlet, 1999; Miller, 1993; Pechenik et al., 1996, 2002; Thiyagarajan, Harder, & Qian, 2003; Thiyagarajan et al., 2005). Previous studies have also revealed a correlation between initial body size and mortality, with smaller individuals experiencing higher rates of mortality than larger EBP individuals (Moran, 1999; Phillips, 2002, 2017; Thiyagarajan et al., 2002). Those studies have suggested the relationship between body size and mortality may be due to increased vulnerability to predation, competition, or abiotic stressors, but it might also be due to insufficient initial energy reserves directly causing EBP mortality. While these indirect lines of evidence are consistent with the hypothesis that depleted initial energy reserves directly cause EBP mortality, the hypothesis remains untested.

**FIGURE 1:**
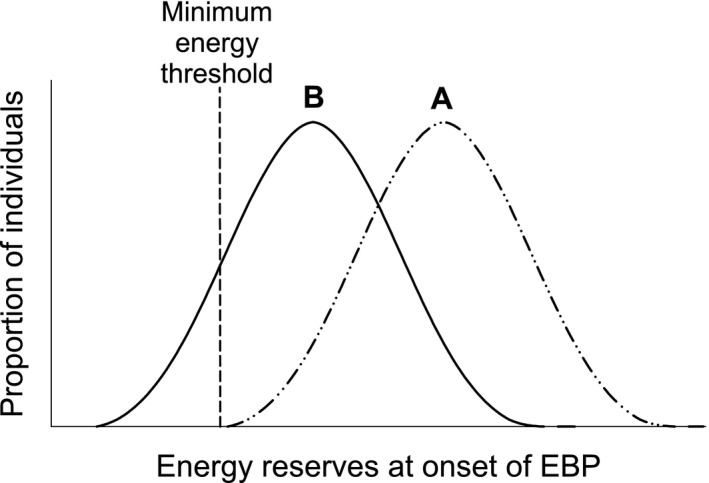
Two hypothetical frequency distributions (bell curves) of energy reserves among individuals at the onset of the EBP. The dashed vertical line represents a minimum energy threshold required for EBP survival

For the depleted initial energy hypothesis to be correct, there must be variation in initial energy reserves, with some individuals having relatively low energy levels. Accordingly, variation in energy reserves at the onset of the EBP among individuals does occur in at least some species, with some individuals beginning the EBP with relatively low energy reserves (Jarrett & Pechenik, 1997; Moran & Emlet, 2001). This variation is largely due to differences in maternal provisioning or in food availability to the larvae, as well as competent larvae of many species transitioning through energetically demanding periods of development prior to the onset of the EBP (Lucas et al., 1979; Thiyagarajan, Harder, Qiu, et al., 2003). The most energetically demanding of these developmental periods is metamorphosis, which involves extensive rearrangement of tissues and loss or replacement of numerous body structures (Bennett & Marshall, 2005; Bryan, 2004; Thiyagarajan et al., 2002; Thiyagarajan, Harder, & Qian, 2003; Wendt, 2000). Additionally, development in some species includes a substantial nonfeeding phase immediately before, during, or following the onset of the EBP (Anderson, 1994; Gosselin & Chia, 1994). During these nonfeeding periods, and until metamorphosis is completed and feeding begins, individuals are solely reliant upon their internal energy stores for resting and active metabolism (Gosselin & Chia, 1994; Lucas et al., 1979; Pechenik et al., 1993). Accordingly, it has been suggested that some portion of individuals may begin the EBP with such critically low energy stores that they are near a minimum threshold necessary to survive (Jarrett & Pechenik, 1997); those individuals would either inevitably die of energy depletion very soon after metamorphosis, or need to quickly feed to replenish energy stores to survive. We illustrate this hypothesis in Figure 1, representing two hypothetical frequency distributions of energy reserves among EBP individuals as well as a theoretical minimum threshold of energy reserves represented by the vertical line. Curve A represents a situation where all individuals have sufficient energy to survive, in which case the depletion of energy reserves would not be a direct cause of EBP mortality. Conversely, curve B represents the current hypothesis, that a proportion of individuals begin the EBP below the minimum energy reserve threshold, and thus inevitably die during or shortly after metamorphosis. In addition, if the hypothesis represented by curve B is correct, then a substantial proportion of EBP individuals would have energy reserves only slightly larger than the minimum threshold; these individuals would need to feed immediately to survive or would otherwise drop below the threshold and die. Thus, a goal of the present study was to test these two hypotheses to establish the role of energy reserves as a direct cause of EBP mortality.

While EBP mortality rates are often very high, they are also highly variable among and within populations (Gosselin & Qian, 1997; Hunt & Scheibling, 1997; Jarrett, 2000; Phillips, 2017). The hypothesis that variation in energy reserves (bell curves in Figure 1) is a cause of the variability in EBP mortality is partly supported by evidence (Jarrett & Pechenik, 1997; Phillips, 2006) of natural variation among individuals in the amount of energy reserves present at the onset of the EBP. Internal energy reserves can vary depending on the source of energy, and in some cases upon the quantity of food available prior to the beginning of the EBP. For species with planktotrophic larvae, EBP energy reserves originate primarily from particulate food consumed during their larval phase. For these species, energy reserves can vary among individuals depending on the quantity and quality of food they encounter (Jarrett, 2003; Moran & Manahan, 2004), whether they transition through a nonfeeding phase, and whether they encounter conditions that delay settlement (Pechenik et al., 1993; Phillips, 2002; Thiyagarajan, Harder, & Qian, 2003; Thiyagarajan et al., 2007). For direct developing species, EBP energy reserves originate entirely from maternal provisioning and thus may vary with the condition of the maternal parent (Moran & McAlister, 2009; van der Sman et al., 2009) or with the genetic programming of the population or species (Allen & Marshall, 2013; Rivest, 1983). In some cases, unfertilized nurse eggs are provided to the embryos to consume as they develop; for these species, energy levels can further vary among individuals of the same cohort depending on the quantity of nurse eggs consumed before hatching (Jablonski & Lutz, 1983; Lloyd & Gosselin, 2011; Marko et al., 2014). All these differences, both interspecific and intraspecific, result in individuals beginning the EBP with vastly different amounts of internal energy reserves, and this might play a substantial role in determining which individuals survive through the first few days of independent benthic life. Additionally, any of these factors has the potential to shift the frequency distribution of energy reserves to the left or right, as depicted by the bell curves A and B in Figure 1, or even alter the shape of these curves and thus also affect the proportion of individuals falling below the minimum threshold for survival.

For individuals starting the EBP with only slightly more internal energy than the minimum threshold, rapidly accessing a new food source to replenish their energy reserves could be vital to their survival. A delay in feeding at this point might drop them below the threshold and thus push them beyond their “point of no return” (commonly referred to as PNR in other publications), a pivotal point beyond which animals are unable to recover even if provided with food (Blaxter & Hempel, 1963; Moran & Manahan, 2004). For individuals that do not feed before this point, both their survivorship and ability to grow are likely to be impaired (Chen et al., 2005; Roberts et al., 2001; Takami et al., 2000). The existence and timing of a point of no return has been studied for the larval phase of several species of intertidal invertebrates (Espinoza et al., 2017; Gebauer et al., 2010; Moran & Manahan, 2004; Yan et al., 2009) but has not been well established for EBP individuals of most species. Determining the starvation point at which an individual's energy reserves are so heavily depleted that they are unable to recover may be useful for understanding how energy reserves impact EBP survivorship and growth.

The main purpose of this study was to determine the role of energy content in regulating survivorship during the critical first days of independent benthic life, thus helping to better understand the mechanisms controlling EBP mortality, and thus recruitment, of benthic invertebrates. The first specific objective of the study was (1) to establish whether energy reserves of EBP individuals could be experimentally controlled by starvation; this was addressed by determining the relationship between total organic content of EBP individuals and the duration of starvation they had experienced. This was followed by two additional specific objectives: (2) to test the hypothesis (Figure 1, curve B) that a large proportion of individuals begin the EBP with critically low energy stores such that they either die or require rapid access to food to survive the first days of independent benthic life, and (3) to examine the effect of delayed feeding on the performance (growth and survival) of EBP invertebrates over a period of several weeks.

## 2 METHODS

### 2.1 Study species

This study examined the role of initial energy reserves on survivorship in six species of benthic intertidal invertebrates. All animals used in this study were from wild populations and had developed through the embryonic and planktonic larval phases in the field. Species were selected based on sufficient availability of early benthic phase (EBP) specimens in the field. All six species are abundant on the west coast of Vancouver Island, Canada. The barnacles *Balanus glandula* Darwin 1854 and *Chthamalus dalli* Pilsbry 1916, as well as the mussel *Mytilus trossulus* Gould 1850, colonize exposed hard substrata in the mid to high intertidal zone. *Nucella ostrina* Gould 1852 and *Nucella lamellosa* Gmelin 1791 are predatory snails that inhabit the mid‐intertidal zone. Finally, *Petrolisthes cinctipes* Randall 1840 and *P. eriomerus* Stimpson 1871 are small (~2 cm) porcellanid crabs that inhabit cryptic habitats, primarily the undersides of large rocks (Jensen, 1989), in the low to mid intertidal zone; EBP individuals of these two species could not be readily distinguished, so these were grouped together and designated as *Petrolisthes* spp.

These species have different sources and quantities of energy reserves at the beginning of the EBP. *N. ostrina* and *N. lamellosa* are lecithotrophic and thus depend entirely on maternal provisioning for EBP energy reserves. The four other species, however, have planktotrophic larvae, and a large portion of their EBP energy reserves come from the food they consume during the larval stage. Additionally, *B. glandula* and *C. dalli* have a nonfeeding cyprid larval stage prior to metamorphosis, and thus use up some energy stores prior to the EBP, whereas *M. trossulus* and *Petrolisthes* spp. are able to feed up to and throughout metamorphosis. By including species with different developmental histories, this study explored how EBP survivorship is impacted by energy reserves across species with different modes of energy acquisition prior to entering the EBP.

### 2.2 Study site and collection of animals

**FIGURE 2:**
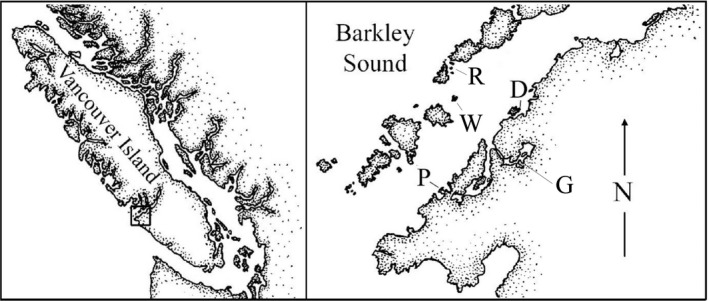
Map of field sites in Barkley Sound near Bamfield, British Columbia: Ross Islets (R), Wizard Islet (W), Prasiola Point (P), Dixon Island (D), and Grappler Inlet (G). Map modified from Gosselin and Chia (1995)

Research was conducted at the Bamfield Marine Sciences Centre (BMSC) on the west coast of Vancouver Island from May to September of 2019. Animals were collected from five rocky intertidal shore sites located in Barkley Sound (Figure 2). Sites were selected based on the availability of EBP animals of each species. Collected animals were brought to and maintained at BMSC, where all experiments were conducted.

Juvenile barnacles (*B. glandula* and *C. dalli*) were collected as described by Sandee et al. (2016) and Hamilton & Gosselin (2020). Small rocks (5–10 cm diameter) were gathered from the intertidal zone and brought to the laboratory where all small settlers were dislodged under a dissecting microscope, and a perimeter was marked on the rock around the cleared area with nail polish. Marked rocks were then returned to the field and left for 48–72 hr (4–6 tidal cycles) to allow for new settlement, after which rocks were recovered and examined for newly settled barnacles. At that age, barnacles would still be reliant upon energy reserves obtained during the larval phase, as juvenile barnacles do not begin to feed until 2–5 days postmetamorphosis (Rainbow & Walker, 1977). Ripe egg capsules of both *Nucella* species (*N. ostrina* and *N. lamellosa*) were collected from intertidal substrata using fine‐tipped forceps as in Gosselin and Chia (1995). Egg capsules were returned to the laboratory and held in small cages in aerated seawater for 72 hr, after which all newly hatched juvenile snails were removed and retained for experiments. EBP *M. trossulus* were extracted from tufts of the filamentous algae *Cladophora columbiana*. Extracted mussels were measured and only recently settled juveniles measuring 250–600 μm shell length (Phillips, 2017) were retained for experiments. *Petrolisthes spp*. megalopae were collected from the underside of large rocks in the intertidal zone. Megalopae were carefully removed with a pair of soft insect forceps and transported to BMSC in small containers. All animals were returned to the laboratory within 3 hr of collection.

### 2.3 Procedures for rearing EBP individuals without food

To experimentally control the energy reserves of EBP individuals, most experiments in this study involved rearing EBP individuals without food. In all experiments described below, the following procedures were used to rear EBP individuals of each species while ensuring they did not have access to food. All animals were held in seawater filtered to 1.0 μm to ensure no food particles were available for feeding. *M. trossulus*, however, can capture particles <1.0 μm (Strohmeier et al., 2012), so filtered water used for this species was also autoclaved and then vacuum‐filtered to 0.45 μm. Rocks containing newly‐settled barnacles were kept in 1 L containers, each with an air stone for aeration. All other animals were kept in small cages (1.5–600 ml) with mesh‐covered cut‐out windows to allow for water flow while preventing escapement. Water was maintained at 15–17°C, which approximates average sea surface temperatures during the summer months (Iwabuchi & Gosselin, 2019). Filtered water was replaced every second day, and holding containers and cages were rinsed and scrubbed to prevent the growth of algae.

### 2.4 Relationship between organic matter content in EBP individuals and duration of starvation

To determine whether starvation could be used as a method for predictably depleting energy reserves in EBP individuals, we tested the hypothesis that energy reserves decrease as a function of the duration of starvation. This experiment was carried out with *N. ostrina* hatchlings, and total organic matter (OM) was measured by ash‐free dry weight (AFDW). AFDW is an effective, albeit simple, indicator of energy content (Moran & McAlister, 2009). It does not, however, reflect the energetic value of any single type of organic matter, such as triacylglycerols (Podolsky, 2002), which are the primary source of metabolizable energy for marine invertebrates (Sewell, 2005; Whitehill & Moran, 2012). Thus, it is assumed that the first OM to be depleted by starved EBP individuals will be these energetic lipids, followed by other forms of OM, resulting in a progressive decrease in AFDW.

Only intermediate‐sized *N. ostrina* hatchlings (1.11–1.40 mm shell length) were used in this experiment. Hatchlings were kept in 50 ml cages, with 100–200 individuals in each cage. Hatchlings were maintained without food following the procedure stated above. Every 10 days, beginning immediately after hatching, samples of 52–60 unfed *N. ostrina* hatchlings were removed from the holding cages. These were briefly rinsed in distilled water to remove adhering salts and then stored at −80°C. AFDW of each sample was obtained using a procedure adapted from Pechenik et al. (1993). For each sample of 52–60 frozen snails, the sample (all snails together) was placed in a preweighed foil pan and dried at 65°C for 48 hr. After drying, the pan was weighed on a Fisher Scientific accuSeries balance with an instrument precision of ±0.01 mg to quantify total dry weight and then placed in a muffle furnace at 500°C for 5 hr. The pan was then placed in a sealed desiccator containing silica gel desiccant for 20 min to cool, and then reweighed to determine ash weight (i.e., the weight of the sample without the OM). AFDW, a measure of OM content of each sample, was calculated by subtracting the ash weight from the initial dry weight. Ash weight consisted almost entirely of the shell, and shell weight was not expected to decline with starvation, so ash weight was used as an indicator of initial snail body mass prior to starvation. The OM estimate (AFDW) was therefore divided by the ash weight to obtain the OM:ash weight ratio; this ratio standardized results for all snails, accounting for any minor differences in initial body size among individuals. This experiment was carried out twice, using two different batches of hatchlings. Batch 1 was collected from Ross Islets on 17 July 2019, and Batch 2 was collected from Prasiola Point on 26 July 2019.

### 2.5 Ability of EBP individuals to survive exclusively on their initial energy reserves

#### 2.5.1 Survivorship of EBP individuals unable to replenish energy reserves

This experiment determined, for each of the six species, the proportion of individuals that begin the EBP with energy reserves close to the minimum threshold required for survival. EBP individuals were collected on several dates throughout the spring and summer, and each batch of individuals was tested separately as a replicate trial. For each species, 2–8 batches of individuals were tested, each batch consisting of 9–200 individuals (Appendix S1). The number of batches collected for each species and the number of individuals in each batch were dependent on the availability of EBP individuals in the field. All animals were held in 50‐mL cages without food, as described above, for up to 100 days starting immediately after the onset of the EBP. Every 10 days, survival was assessed by examining all individuals for movement under a dissecting microscope. If an animal did not show any signs of movement within 2 min, their tissues were gently contacted with a probe and they were observed for an additional 2 min. If no movement was observed at this point, the individual was recorded as dead and removed from the experiment. The proportion of individuals surviving each 10‐day period was recorded.

#### 2.5.2 Influence of initial body mass on survivorship of EBP individuals unable to replenish energy reserves

There is a strong correlation between body mass and quantity of organic matter among EBP invertebrates (Moran & Emlet, 2001). Specifically, larger individuals have larger stores of lipids (Emlet & Sadro, 2006; Phillips, 2002), which are the primary metabolizable energy source for many intertidal species (Sewell, 2005; Thiyagarajan et al., 2002; Whitehill & Moran, 2012). Thus, body mass was used as a proxy for energy reserves to determine their influence on survivorship. To determine how the ability to survive periods of starvation is influenced by body mass at the start of the EBP, the survivorship of three size classes of newly hatched *N. ostrina* were examined. Most *N. ostrina* egg capsules contain 7–20 fertilized embryos that will hatch into juvenile snails, as well as 500–600 unfertilized nurse eggs that the embryos consume as they develop inside the capsule (Lloyd & Gosselin, 2011; Marko et al., 2014). *N. ostrina* hatchlings consume different numbers of nurse eggs while in the capsule, and therefore, hatchlings range considerably in size upon emergence, with larger hatchlings having consumed more nurse eggs and thus having greater energy reserves than smaller hatchlings. Newly hatched *N. ostrina* were sorted into three shell length (SL) categories: small (0.81–1.10 mm SL), medium (1.11–1.40 mm SL), and large (1.41–1.80 mm SL). These SL measurements were then converted to body mass (wet weight, WW) using a regression equation for *N. ostrina* from Hamilton & Gosselin (2020) (Table 1). Eight batches of hatchlings were collected throughout the spring and summer; each batch was sorted into the three size classes, and each size class of a given batch contained 11–217 hatchlings (Appendix S1). In some batches, certain size classes were not included in this experiment because too few individuals of the size class were available. Hatchlings were held in 50 ml cages without food for up to 70 days, beginning at the time of hatching (i.e., the onset of EBP). Every 10 days, survival was assessed by examining all individuals under a dissecting microscope, as described above, and the number of survivors of each size class was recorded.

**TABLE 1:** Shell lengths (SL) and corresponding body masses (wet weight, WW) for *N. ostrina* hatchlings

Size class	Shell length (SL) in mm	Body mass (WW) in mg
Small	0.81–1.10	0.074–0.192
Medium	1.11–1.40	0.197–0.394
Large	1.41–1.80	0.402–0.833

Note

The conversion equation is: WW =2.98 * log(SL) ‐ 3.84, from Hamilton & Gosselin (2020).

### 2.6 Ability to recover after delayed feeding in EBP invertebrates

The ability of EBP individuals to recover from periods of starvation was examined in three species: *B*. *glandula*, *C. dalli*, and *N. ostrina*. EBP individuals of each species were reared without food, beginning at the onset of EBP, for various durations (0, 10, 20, 30, 40 or 50 days) before being fed for 30 days. Growth during the 30‐day feeding period was determined by measuring the size (rostro‐carinal diameter for barnacles, SL for *N. ostrina*) of each individual at the end of the starvation period (i.e., just before feeding began), and then again at the end of the 30‐day feeding period. The number of individuals that died during the 30‐day feeding period was also recorded for each group. All individuals of the same species were within a narrow size range at the beginning of the experiment. The experimental design was as follows: 3 species X 6 duration of starvation treatments per species X 10–15 individuals per starvation treatment.

During the feeding period, *B. glandula* and *C. dalli* were held in 1‐L aerated containers and fed a mixture of diatoms (*Chaetoceros muelleri* and *C. gracilis*) and flagellates (*Tertaselmis* spp.) that had been reared in the laboratory. Algae were provided at a total concentration of 1x10^6^ cells/mL, determined by counting algal cells with a hemocytometer. Water and algae were changed every second day. To feed *N. ostrina* hatchlings, each individual was placed in a small (1.5 ml) cage with 10 juvenile mussels (*M. trossulus*), a preferred prey species for EBP *N. ostrina* (Gosselin & Chia, 1994). *N. ostrina* feed by drilling a small hole through the shell of their prey with their radula, making it possible to determine whether the hatchling had fed.

To assess the effect of delayed feeding on the performance of EBP invertebrates, individuals were categorized as either recovering (i.e., alive and showing growth) or not recovering (i.e., dead, or alive but with no growth) following the 30‐day feeding period. The percent of individuals that were recovering in each starvation treatment was then calculated for each species. Additionally, the growth (i.e., change in size) of each EBP individual during the feeding period was calculated to assess the effects of delayed feeding on the ability to resume growth when food becomes available.

### 2.7 Data analysis

In all experiments, survivorship data followed a binomial distribution. All survivorship data were analyzed using generalized linear models (GLM) with a binomial link function to determine the starvation LD_50_ for each species, and the % survivorship after 5‐day starvation and the starvation LD_20_ for each size class of *N. ostrina*. The fit of each GLM model was tested by comparing the residual deviance to the residual degrees of freedom, which indicated goodness of fit for each model (i.e., residual deviance / residual *df* < 1). One‐way ANOVA and the Tukey HSD multiple comparisons test were used to compare the % survivorship after 5‐day starvation and the starvation LD_20_ among the three size classes of *N. ostrina* hatchlings. The Shapiro–Wilk normality test and the Bartlett test of homogeneity of variances were used to assess the assumptions of ANOVA. All statistical analyses were conducted using R statistical software (version 3.5.0) (R Core Team, 2018).

## 3 RESULTS

### 3.1 Relationship between organic matter content in EBP individuals and duration of starvation

**FIGURE 3:**
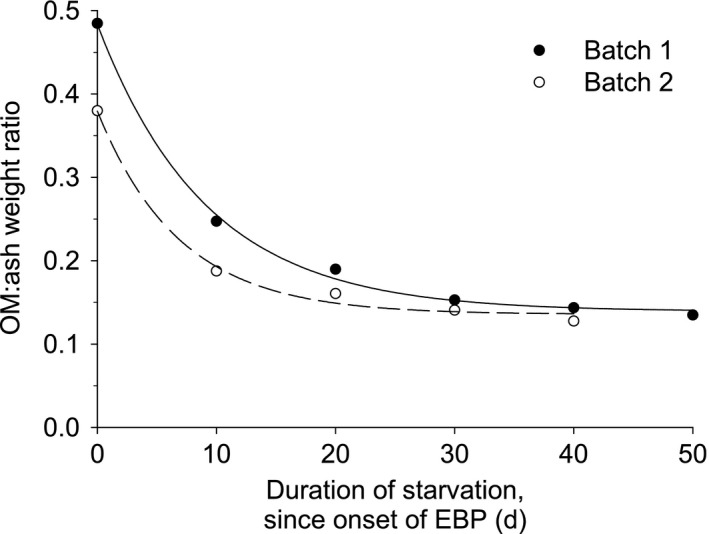
Relationship between OM:ash weight ratio and the duration of starvation, starting at the onset of the EBP, for *Nucella ostrina* hatchlings. OM is used as an indicator of total energy content and ash weight is used as an index of initial total body mass. Each value represents a combined sample of 52–60 individuals. Each batch represents a single collection of hatchlings from the field

The relationship between OM:ash weight ratio and duration of starvation starting at the onset of the early benthic phase (EBP; Figure 3) was effectively described by an exponential decay model:$y=y0+a*e^{‐bx}$ where *y0* is the intercept, *a* is the lower limit reached for *y*, *x* is the duration of starvation, and *b* is the decay coefficient. The OM:ash weight ratio of *N. ostrina* was significantly related to the duration of starvation in both batches of *N. ostrina* hatchlings (Batch 1: *F* = 583.95, *df* = 6, *R* ^2^ = 0.996, *p* <.001; Batch 2: *F* = 175.45, *df* = 5, *R* ^2^ = 0.989, *p* =.006). Based on this analysis, approximately half the OM content of an individual was utilized by day 10 of starvation (Batch 1: 51.05%, Batch 2:​ 49.35%).

### 3.2 Ability of EBP individuals to survive exclusively on their initial energy reserves

#### 3.2.1 Survivorship of EBP invertebrates unable to replenish energy reserves

**FIGURE 4:**
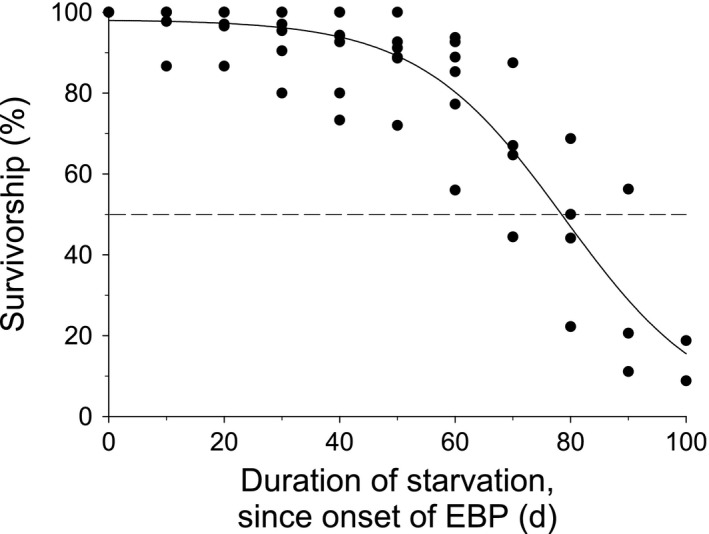
Percent survivorship of starved *B. glandula* as a function of the duration of starvation, starting at the onset of the EBP. The point where the curve intersects the dashed line represents the starvation LD_50_

Survivorship was impacted by the duration of starvation experienced by the individuals (e.g., *B. glandula*, Figure 4). The relationship, assessed using GLM with a binomial link function, was significant in four of the six species (Table 2). For *M. trossulus* and *Petrolisthes spp*., the relationship was not quite significant, likely due to the modest sample sizes, as only two batches of individuals were collected for each of these species, whereas 5–9 replicate batches were examined in each of the other four species. The trends of decreasing survivorship as a function of duration of starvation for *M. trossulus* and *Petrolisthes spp*. were nevertheless similar to those of the other four species (Appendix S1).

**TABLE 2:** Starvation LD_50_ and associated standard error (SE) from generalized linear model (GLM) analysis of survival as a function of duration of starvation for each of the 6 species

Species	Starvation LD_50_ (d)	SE	z statistic	*df*	p
*B. glandula*	78.4	7.59	−3.40	62	<0.001
*C. dalli*	69.3	7.20	−3.23	42	0.001
*N. ostrina*	55.9	9.66	−2.56	51	<0.001
*N. lamellosa*	27.9	4.75	−2.99	30	0.003
*M. trossulus*	58.3	14.28	−1.76	16	0.079
*Petrolisthes spp*.	50.8	5.70	−1.59	12	0.113

Note

The z statistic represents the strength of the relationship between the % survivorship and the duration of starvation; *df* =degrees of freedom.

**FIGURE 5:**
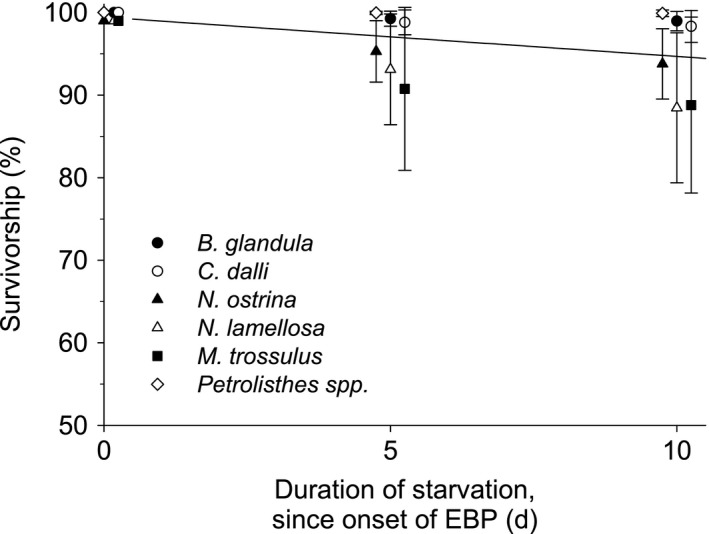
Survivorship of individuals starved for the first 10 days since the onset of EBP for six benthic intertidal species. Each point represents the average % survivorship and associated standard errors calculated from GLMs for each species (Table 1)

The duration of starvation that is lethal to 50% of individuals (starvation LD_50_), as determined from the above GLM analyses, varied substantially among species (Table 2). Starvation LD_50_ ranged from 27.9 ± 4.75 days (average ± SE) for *N. lamellosa* to 78.4 ± 7.59 days for *B. glandula*. When survivorship of all six species during the first 10 days of starvation was combined in a single regression analysis, the percent survivorship declined significantly, but moderately (Linear regression: *F* = 7.589, *n* = 6, R^2^ = 0.26, *p* =.012) (Figure 5). As a result, survivorship remained high throughout these first 10 days. After 5 days of starvation, average survivorship was >90% in all species; after 10‐day starvation, average survivorship was still >90% in four of the six species (*B. glandula*, *C. dalli*, *N. ostrina*, and *Petrolisthes spp*), and >85% for the remaining two (*N. lamellosa* and *M. trossulus*).

#### 3.2.2 Influence of initial body mass on survivorship of EBP individuals unable to replenish energy reserves

Survivorship was significantly affected by the duration of starvation for each batch of all three size classes of *N. ostrina* hatchlings (Table 3). The shape of this relationship, however, differed between size classes. The impact of energy depletion on survivorship of *N. ostrina* in each size class during the critical first days of the EBP was examined using two different approaches: (1) determining the proportion of individuals that survived 5 days of starvation after hatching, and (2) determining the number of days of starvation required to cause 20% mortality of hatchlings (starvation LD_20_). The starvation LD_20_ was used instead of the LD_50_ because some of the Medium and Large size class batches did not reach 50% mortality during the time frame of the experiment.

**TABLE 3:** Results of the GLM analysis of percent survivorship as a function of duration of starvation for each size class from each batch of *N. ostrina* hatchlings

Batch	Size class	*n*	Estimate	SE	z value	*df*	p
1	Small	13	−0.056	0.010	−5.37	8	<0.001
	Medium	29	−0.034	0.015	−2.34	5	0.020
2	Small	106	−0.092	0.006	−15.78	7	<0.001
	Medium	90	−0.078	0.007	−11.20	7	<0.001
	Large	217	−0.036	0.004	−8.22	7	<0.001
3	Medium	37	−0.088	0.012	−7.14	4	<0.001
	Large	56	−0.045	0.006	−7.16	7	<0.001
4	Large	59	−0.050	0.007	−6.87	6	<0.001
5	Small	11	−0.108	0.028	−3.87	5	<0.001
	Medium	25	−0.033	0.012	−2.67	5	0.008
	Large	15	−0.036	0.018	−2.03	5	0.042
6	Small	57	−0.088	0.009	−9.05	5	<0.001
	Medium	152	−0.058	0.006	−10.17	5	<0.001
	Large	76	−0.051	0.009	−5.99	5	<0.001
7	Small	34	−0.102	0.014	−7.57	5	<0.001
	Medium	115	−0.042	0.008	−5.33	5	<0.001
	Large	85	−0.080	0.016	−5.12	5	<0.001
8	Small	68	−0.077	0.014	−5.38	4	<0.001
	Large	34	−0.105	0.034	−3.05	4	0.002

Note

Z statistics indicate the fit of the relationship between duration of starvation and % survivorship; *df* =degrees of freedom. The binomial link function was used in all GLMs.

**FIGURE 6:**
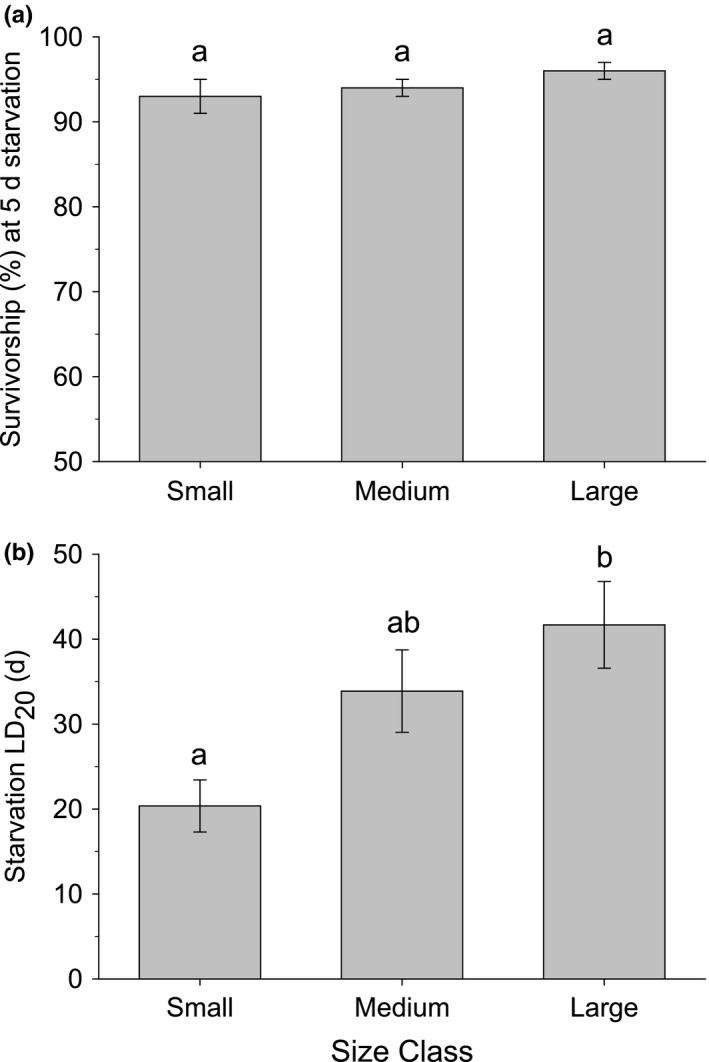
Effect of initial body size of *Nucella ostrina* hatchlings on (a) the survivorship at 5 days and (b) the starvation LD_20_. Small = 0.80–1.10 mm shell length; Medium = 1.11–1.40 mm shell length; Large = 1.41–1.80 mm shell length. Each mean value and associated standard error are calculated from generalized linear models (GLM) for 6–7 batches of each size class. Different letters above the bars indicate values that were significantly different based on Tukey HSD tests (*p* <.05)

The three size classes survived equally well without food in the short‐term, but their fate eventually differed as time without food became extended. Survivorship after 5 days of starvation did not differ significantly among the three size classes (one‐way ANOVA: *F* = 1.207, *df* = 16, *p* = .325), with survivorship ranging from 93% to 96% (Figure 6a). The starvation LD_20_, however, did differ significantly among size classes (one‐way ANOVA: *F* = 5.718, *df* = 16, *p* = .013) (Figure 6b). The LD_20_ for the Small size class was significantly shorter than for the Large size class (Tukey HSD: *p* = .010), whereas the LD_20_ for the Medium size class was not significantly different from either the Small (*p* = .132) or the Large (*p* =.453) size classes.

### 3.3 Ability to recover after delayed feeding for EBP invertebrates

**FIGURE 7:**
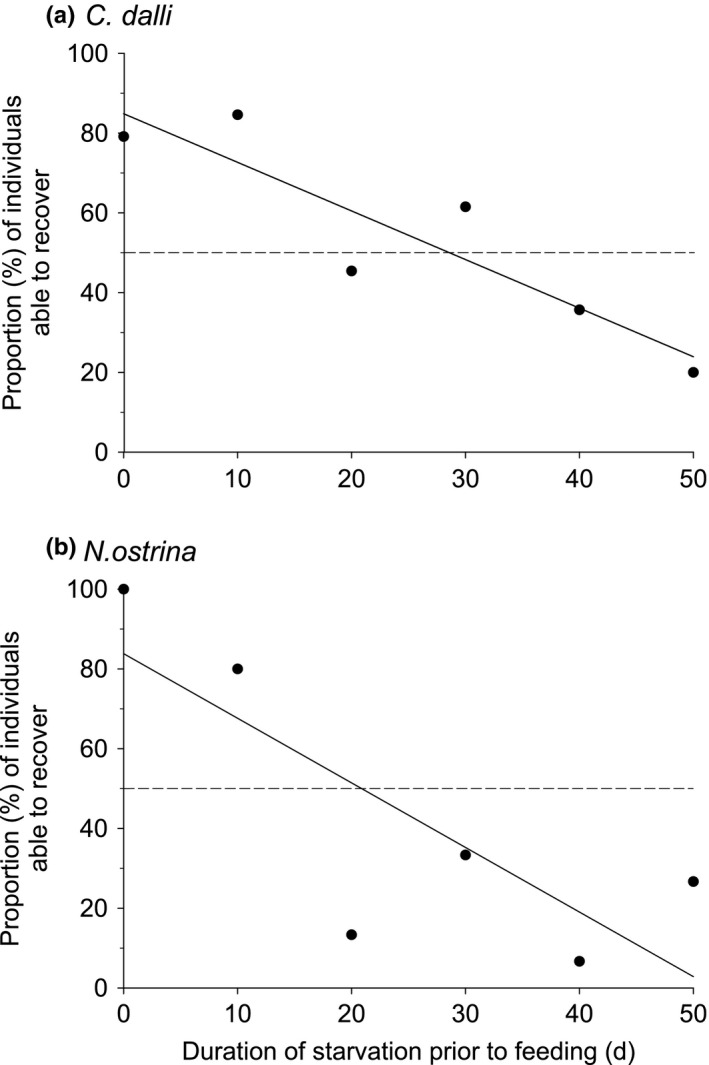
Percent of individuals able to recover, as a function of duration of starvation in (a) *C. dalli* and (b) *N. ostrina*. Equations of the regression lines: (a) *y* = 84.8758–1.2184x, and (b) *y* = 83.8095–1.6190*x*. The point where the model intersects the dashed line indicates the time of starvation resulting in only 50% of individuals being able to recover when fed

The duration of starvation experienced before being offered food had a significant or nearly significant effect on the percent of individuals able to recover (i.e., survive and grow during the 30 days feeding period) for *C. dalli* (Linear regression: *F* = 17.56, *n* = 6, R^2^ = 0.81, *p* =.014) and *N. ostrina* *(F* = 7.08, *n* = 6, R^2^ = 0.64, *p* =.056) (Figure 7). The duration of starvation resulting in only 50% of individuals being able to recover (i.e., the recovery LD_50_) was >20 days in both species, but was somewhat longer in *C. dalli* than in *N. ostrina* (*C. dalli*: 28.6 ± 4.2 days (SE); *N. ostrina*: 20.9 ± 6.6 days).

In *B. glandula,* the ability of individuals to recover was not significantly affected by the duration of starvation they experience (Linear regression: *F* = 0.512, *n* = 6, R^2^ = 0.11, *p* =.512). Once *B. glandula* individuals were provided with food after any period of starvation, very few deaths were observed (3 deaths, each from a different starvation group, out of a total of 88 individuals), and all surviving individuals exhibited some growth during the 30‐day feeding period. Thus, to analyze the effect of starvation on recovery of *B. glandula*, the average growth throughout the 30‐day feeding period was calculated for each treatment (i.e., each duration of starvation) in order to assess the effect of starvation on their ability to grow once food becomes available. Additionally, each individual was measured at the end of the starvation period just prior to feeding, and an average shell diameter was calculated for each treatment group to assess if any growth occurred during the starvation period. The three individuals that died during the 30‐day feeding period were excluded from this analysis.

**FIGURE 8:**
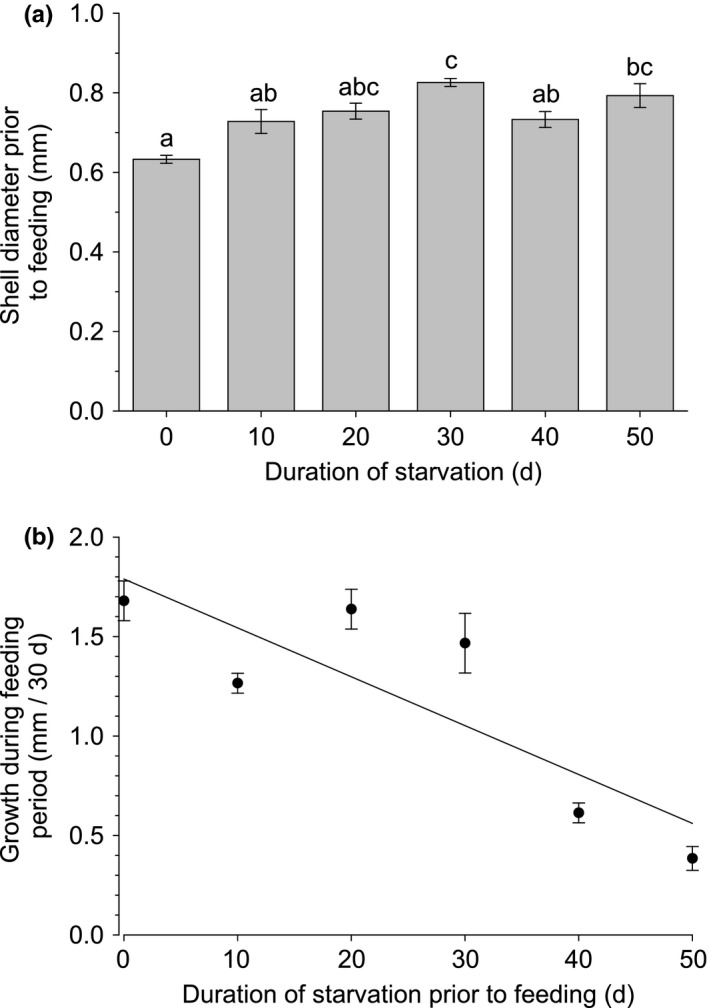
Effect of duration of starvation on (a) shell diameter prior to feeding, and (b) subsequent growth of EBP *B. glandula*. Values represent average shell diameter ± SE at the end of the period of starvation (a) and average growth ± SE during the 30‐day feeding period (b). Different letters above error bars on the bar graph indicate values that were significantly different. Equation of the regression line in (b): *y* = 1.76033–0.023276*x*

Shell diameter at the end of the starvation period differed among treatment groups (Figure 8a) (one‐way ANOVA: *F* = 7.90, *df* = 74, *p* <.001). This indicates that even in the absence of particulate food, EBP *B. glandula* deposited some new calcium carbonate into their shells, thus increasing their shell diameter. However, the increase in shell diameter was moderate (up to 0.15 mm, or 22%, increase) and significant increases did not occur beyond 20 days of starvation. In contrast, growth during the 30‐day feeding period was significantly and strongly affected by the duration of starvation experienced (Figure 8b) (Linear regression: *F* = 65.93, *n* = 79, R^2^ = 0.46, *p* <.001). Individuals starved for longer periods of time grew at a substantially slower rate than those starved for a shorter period once particulate food was introduced.

## 4 DISCUSSION

### 4.1 Relationship between organic matter content in EBP individuals and duration of starvation

Energy content in early benthic phase (EBP) *N. ostrina* declined rapidly, and in a predictable way, as a function of duration of starvation. The OM:ash weight ratio, a proportional estimate of energy content (relative to initial body size), significantly decreased as the period of starvation increased. The depletion of energy content was particularly rapid in the first weeks of starvation; the OM:ash weight ratio dropped by half after only 10 days of starvation, and decreased by an additional 19% by day 20. In both batches of hatchlings, the OM:ash weight ratio levelled off by day 40 at ~0.14, in other words when the mass of remaining organic matter in the body was 14% of the mass of the inorganic material, the latter mostly consisting of the shell. This continuous reduction in OM content is consistent with hatchlings progressively using up their available energy reserves when not able to feed, and confirms the assumption that AFDW is an effective measure of energetic lipids. The minimum OM:ash weight ratio of 0.14 is likely determined by the amount of organic matter that is difficult to use as an energy source by the hatchlings. Almost all organic compounds that can readily be metabolized to release energy, mainly energetic lipids such as triacylglycerols (Gosselin et al., 2019; Lee et al., 2006; Sewell, 2005; Thiyagarajan et al., 2002; Whitehill & Moran, 2012), would thus be utilized within 40–50 days of the onset of the EBP if starved, leaving compounds such as structural lipids and proteins as the bulk of the remaining organic matter. Since energy content predictably decreases as a function of the duration of starvation, this finding confirmed that duration of starvation is a useful proxy for metabolizable energy content at the onset of the EBP.

### 4.2 Ability of EBP individuals to survive exclusively on their initial energy reserves

#### 4.2.1 Survivorship of EBP invertebrates unable to replenish energy reserves

Short‐term starvation of individuals from the onset of the EBP did not have the same implications for survivorship as longer‐term starvation. Unsurprisingly, for all six species in this study, starvation eventually caused high mortality. However, a large proportion of individuals of all species were capable of surviving prolonged periods of starvation; in 5 of the 6 species, more than 50% of individuals were still alive after 50 days of starvation, and some individuals survived starvation for more than 100 days. In addition, survivorship during starvation decreased gradually within each species, suggesting considerable variation in either the amount of initial energy reserves or in metabolic rate among individuals of a given species at the onset of early benthic life. Together, these results indicate that a large proportion of EBP individuals can survive for remarkably extended periods of time solely on the internal energy reserves obtained either by larval feeding in planktotrophic species or from maternal provisioning in direct‐developing species.

The most significant finding of this study was that all six species of invertebrates were highly tolerant of starvation in the short‐term, that is, the first few days of the EBP. Survivorship was minimally impacted by starvation during the first 10 days of the EBP, with three species experiencing <2% mortality, and the remaining three species experiencing only 6%–12% mortality through this time period. This finding is not consistent with the starting hypothesis that a substantial proportion of individuals would die in the first few days of starvation due to depleted energy stores (Figure 1, curve B). Rather, these low rates of mortality suggest that for each of the examined species, very few individuals, if any, enter the EBP with such critically low energy reserves that they are below or only slightly above the minimum threshold needed to survive.

This finding of very low mortality of starved individuals during the first days of early benthic life is surprising. Many species of intertidal invertebrates have extremely high mortality rates in the field, especially during the first 10 days of the EBP, often ranging from 30% to 100% (Gosselin & Qian, 1997; Hunt & Scheibling, 1997). More specifically, high morality during the first 3–10 days of early benthic life have been reported for wild populations of a variety of intertidal taxa, some of which were included in the present study: barnacles (Gosselin & Jones, 2010; Gosselin & Qian, 1996; Jenewein & Gosselin, 2013a; Shanks, 2009), gastropods (Moran & Emlet, 2001; Spight, 1975), mussels (Bownes & McQuaid, 2009; Von Der Meden et al., 2012; Phillips, 2017), and crabs (Spitzer et al., 2003). Several studies have hypothesized that the high rate of mortality during the first few days of the EBP might be due in part to a substantial portion of individuals having insufficient internal energy reserves at the beginning of the EBP (Gosselin & Qian, 1997; Jarrett & Pechenik, 1997; Phillips, 2002, 2017; Thiyagarajan et al., 2002, 2007). The present study, however, demonstrated that under controlled conditions, where individuals were isolated from other mortality factors (e.g., predation, competition, environmental stressors), survivorship remained high during the first weeks of the EBP even when animals were starved and thus relied solely on existing energy reserves present at the onset of the EBP. Interestingly, the impact of starvation on early survivorship was similar regardless of mode of larval development or source of EBP energy reserves. The four species with planktotrophic larvae (*B. glandula*, *C. dalli*, *M. trossulus*, and *Petrolisthes* spp.) as well as the two direct developing species (*N. ostrina* and *N. lamellosa*) all had remarkably high survivorship throughout the first 10 days of EBP when starved. This pattern was also unaffected by whether the larvae continue to feed through to competence and settlement or experience some period of pre‐EBP starvation as a result of having a nonfeeding phase. Together, these findings fail to reject the theoretical null hypothesis depicted by curve A in Figure 1, revealing that depleted energy reserves are not a significant cause of the high mortality rates observed during the first weeks of the EBP.

#### 4.2.2 Influence of initial body mass on survivorship of EBP individuals unable to replenish energy reserves

Differences in starvation tolerance among the three size classes of newly hatched *N*. *ostrina* depended on the temporal scale at which these were examined. When considering longer‐term impacts, the time required to reach 20% mortality (i.e., starvation LD_20_) differed among size classes, with small individuals reaching this point an average of 23 days earlier than their larger counterparts. This is consistent with the idea that smaller individuals have a smaller reserve of metabolizable energy and reach a minimum energy threshold more quickly during periods of starvation than larger individuals. However, the impact of size in determining starvation tolerance was subtle, as a difference was only observed between the largest and smallest size classes; the intermediate size class did not differ from either of the other size classes in their ability to tolerate starvation. The advantage bestowed by the larger energy reserve of large hatchlings was also moderate; even small individuals were able to survive for extended periods of time without replenishing energy stores.

The modest long‐term energetic advantage of a larger body size was not apparent on a shorter time scale: There was no significant effect of hatchling size on survivorship during the first 5 days of the EBP. This indicates that even the smallest size class of *N. ostrina* hatchlings have sufficient energy upon hatching to survive through the first 5 days without replenishing their energy stores, consistent with the null hypothesis that nearly all individuals enter the EBP with sufficient energy reserves (Figure 1, curve A). This is not entirely surprising for *N. ostrina*, as hatchlings of this species do not begin feeding until 3–10 days after hatching (Gosselin & Chia, 1994). These findings nevertheless further demonstrate that depletion of energy stores is not a significant cause of EBP mortality.

Offspring size is often a good indicator of offspring fitness; indeed, this is a central tenet of life history theory (Roff, 1992; Sinervo, 1990; Stearns, 1992). For benthic invertebrates, larger larval size often translates into larger EBP size, which in turn leads to greater performance (survival and growth) during the EBP (Emlet & Sadro, 2006; Jarrett & Pechenik, 1997; Marshall & Keough, 2004; Phillips, 2002; Thiyagarajan, Harder, Qiu, et al., 2003). While many of these studies have focused on the larval stage, very few studies have examined the relationship between offspring size and offspring performance after the onset of EBP. The present study found no significant difference in short‐term survivorship among starved individuals of different initial body sizes, and only modest differences in long‐term survivorship. These findings are not dissimilar to those of Moran and Emlet (2001), who found increased survivorship for larger hatchlings in longer outplant experiments (36–54 days), but no significant difference in shorter duration outplants (9 days). This indicates that the short‐term response to starvation and energy depletion is independent of the size of recently hatched individuals.

In populations of many benthic species, larger offspring do tend to outperform their smaller counterparts; larger offspring grow more quickly (Marshall et al., 2003; Moran & Emlet, 2001; Thiyagarajan, Harder, Qiu, et al., 2003; Torres et al., 2016), reach maturity earlier (Marshall et al., 2003; Moran & Emlet, 2001), and often have lower rates of mortality (Emlet & Sadro, 2006; Phillips, 2002; Thiyagarajan, Harder, Qiu, et al., 2003) than smaller offspring. The results of the present study, however, indicate that the larger energy reserve of a larger body size does not lead to greater intrinsic performance during the first days of the EBP. Rather, having a larger body size may provide other benefits that allow for greater success. A larger body size may allow individuals to handle and consume prey more efficiently and thus grow at a faster rate (Moran & Emlet, 2001; Palmer, 1990). Being larger, or being able to grow quickly to reach larger body sizes, also decreases risk of predation (Bashevkin & Pechenik, 2015; Paine, 1976; Rumrill, 1990) and vulnerability to desiccation (Gosselin, 1997) by reaching a size refuge.

### 4.3 Ability to recover after delayed feeding for EBP invertebrates

Although EBP individuals could survive extended periods of starvation, their ability to recover once access to food was regained was negatively affected by the duration of starvation to which they were exposed at the onset of the EBP. The effect, however, was expressed differently among species. In *C. dalli* and *N. ostrina*, the proportion of individuals able to survive and begin to grow once food was introduced decreased with increasing duration of starvation. Conversely, in *B. glandula* the proportion of individuals able to recover was not impacted by the duration of starvation, but the rate of growth throughout the 30‐day feeding period decreased significantly as the duration of starvation increased.

In *C. dalli* and *N. ostrina*, recovery was impacted by starvation, and in both species, the duration of starvation that resulted in only 50% of individuals able to recover was considerably shorter than the starvation LD_50_, found in the previous experiment. Thus, although individuals are able to survive on internal energy stores for extended periods of time, they may still be destined to die if unable to replenish those energy stores much sooner than the LD_50_. One possible explanation for the difference between the starvation LD_50_ and the starvation period resulting in 50% recovery is that extended periods of starvation may cause EBP individuals to lose the capacity to feed by inducing a reabsorption or atrophy of digestive structures (Espinoza et al., 2017; Takami et al., 2000). From that point on, individuals would then continue to deplete all remaining available internal energy reserves but be incapable of efficiently feeding and thus replenishing those energy stores if food were to become available again. We did note that *C. dalli* exhibited behaviors associated with feeding, such as extending and beating their cirri, throughout the 30‐day feeding period even in individuals that did not grow; cirral beating in those individuals most likely served primarily for gas exchange. Similarly, each *N. ostrina*, including those that did not grow, attacked at least one juvenile mussel during the 30‐day feeding period, as evidenced by drill marks observed on the valves of dead mussels. This suggests that although EBP individuals are still capable of exhibiting behaviors associated with feeding, extended periods of starvation may have reduced their ability to digest or absorb nutrients, even if they are able to access food items. Another possible explanation for the inability of individuals to recover from prolonged periods of starvation is that they were unable to extract sufficient energy from consumed food to both replenish the energy they had spent during the starvation period and also begin to grow. In this case, more food, or a longer feeding period, may be required before these animals can fully recover.

The recovery of starved *B. glandula* after regaining access to food differed relative to the other species in this study. Regardless of the duration of starvation experienced in this study, virtually all *B. glandula* individuals showed some ability to recover. The rate of growth throughout the 30‐day feeding period, however, was significantly impacted by the duration of starvation. The 0‐day group, which did not experience any starvation, increased in size five times more during the 30‐day feeding period than the group starved for 50 days. This considerable reduction in growth rate following starvation may have further implications for survivorship, as slower growing individuals will take longer to reach a size refuge, and thus remain susceptible to factors such as competition (Connell, 1961; Pechenik, 1990), predation (Gosselin & Rehak, 2007; Osman & Whitlach, 1995), and desiccation (Hamilton and Gosselin 2020) for a longer period of time.

*B. glandula* also differed from the other species in terms of growth of shell material while being starved (i.e., prior to feeding). During the first 20 days of starvation, some growth was apparent in all groups of EBP *B. glandula*. While growth throughout the starvation period was modest, it indicates that EBP *B. glandula* are capable of depositing new shell material and thus increasing their shell diameter for the first few weeks of the EBP even without feeding and thus without replenishing their energy stores. This suggests that increasing shell size during the EBP is not entirely reliant on obtaining food, nor is it exclusively stimulated by the growth of body tissues. However, the increase in shell diameter in newly settled *B. glandula* that have been without food for any duration was greatly reduced when compared to individuals that had fed.

Finally, in all three species studied in this experiment, an extended period of starvation following the onset of the EBP eventually had a negative impact on the ability to recover, either by directly causing mortality or by impacting growth. Although depletion of energy was not found to be a direct, major source of early mortality among EBP individuals, these findings suggest that EBP individuals with reduced energy reserves would still be at a marked disadvantage for longer‐term survival in the intertidal zone.

### 4.4 Ecological implications

Cohorts of benthic invertebrates in the natural environment suffer extremely high mortality rates during the first days and weeks of the EBP. This study revealed that insufficient energy reserves are not likely a direct cause of this EBP mortality, as had previously been hypothesized (Gosselin & Qian, 1997; Hunt & Scheibling, 1997; Jarrett & Pechenik, 1997; Phillips, 2017). For those individuals that survive through the larval phase and make it to the start of the EBP, energy reserves obtained through larval feeding or maternal provisioning thus appear to be sufficient to sustain the metabolism of the individual through the critical first days of early benthic life. This further indicates that low food availability during the first 5–10 days of the EBP would not be a direct cause of mortality during that time. EBP survivorship in wild populations might nevertheless be impacted by initial energy reserves through indirect effects. Low energy reserves may cause EBP individuals to have low tolerance thresholds to environmental stressors, such as extreme temperatures (Hamilton and Gosselin 2020; Jenewein & Gosselin, 2013b; Miller et al., 2009), desiccation (Foster, 1971; Gosselin & Chia, 1995; Jenewein & Gosselin, 2013a, 2013b; Miller et al., 2009), and low salinity (Qiu & Qian, 1999; Thiyagarajan et al., 2007). If so, this could explain previously reported associations between low energy reserves and high EBP mortality rates in some species (Emlet & Sadro, 2006; Phillips, 2002; Thiyagarajan, Harder, Qiu, et al., 2003). The role of energy levels on stress tolerance thresholds and the possible indirect effects of initial energy reserves on EBP mortality are not well understood and require further investigation.

## CONFLICT OF INTEREST

None declared.

## AUTHOR CONTRIBUTIONS

**Shannon Mendt:** Conceptualization (supporting); Data curation (lead); Formal analysis (lead); Investigation (lead); Methodology (equal); Project administration (supporting); Validation (equal); Visualization (equal); Writing‐original draft (lead); Writing‐review & editing (equal). **Louis Gosselin:** Conceptualization (lead); Data curation (supporting); Formal analysis (supporting); Funding acquisition (lead); Methodology (equal); Project administration (lead); Resources (lead); Supervision (lead); Validation (equal); Visualization (equal); Writing‐review & editing (equal).

## Supporting information

Fig S1

Appendix S1

## Data Availability

Data available from the Dryad data repository (https://doi.org/10.5061/dryad.7d7wm37v4).
