# Supplementary figures and images for: Role of depleted initial energy reserves in early benthic phase mortality of six marine invertebrate species
Source: Ecol Evol. 2021 Jun 6;11(13):8882–96. doi: 10.1002/ece3.7723 (PMC8258192; doi:10.1002/ece3.7723)

A) *B. glandula*

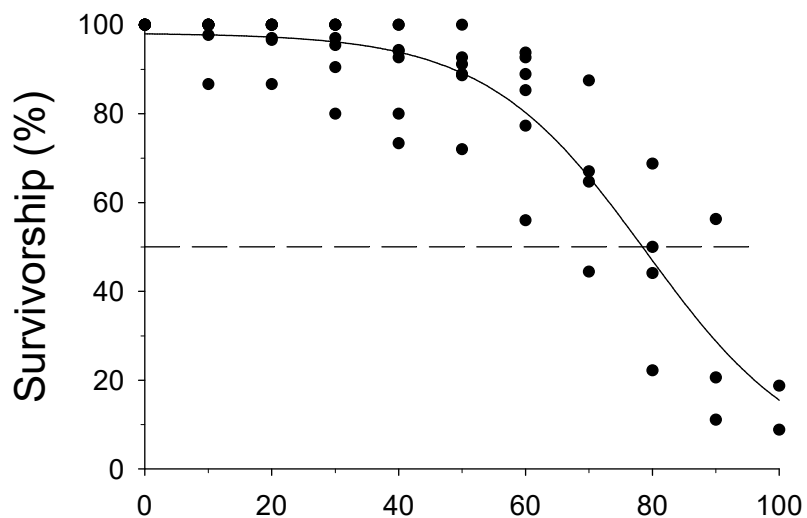

B) *C. dalli*

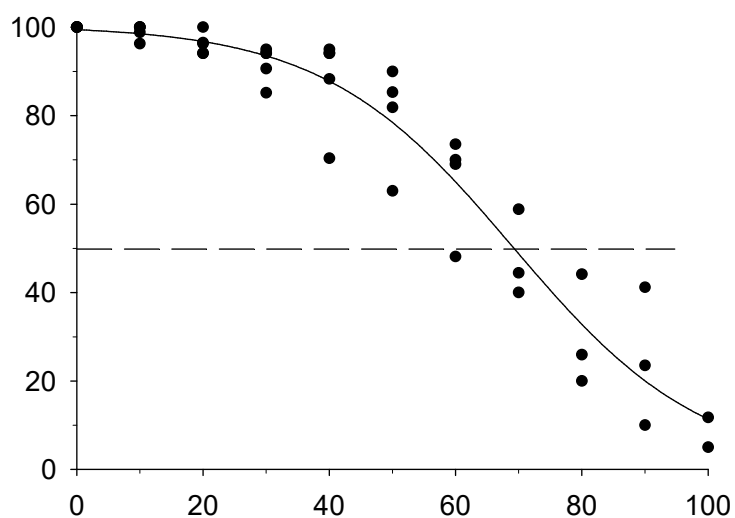

C) *N. ostrina*

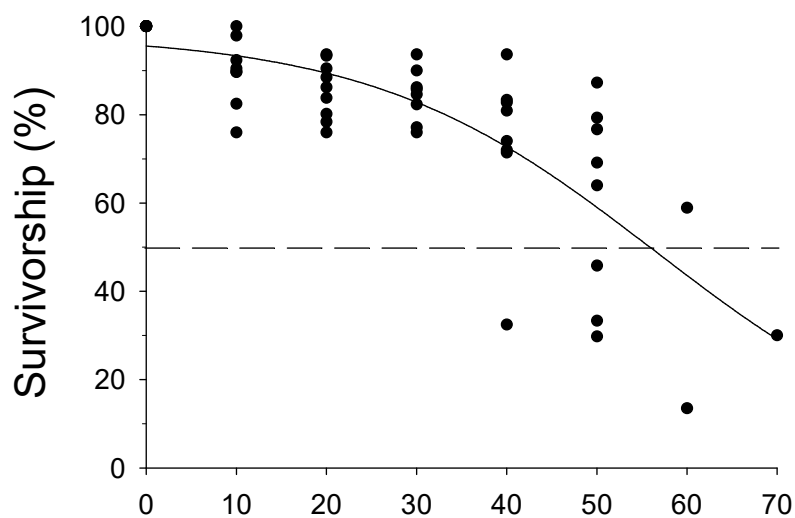

D) *N. lamellosa*

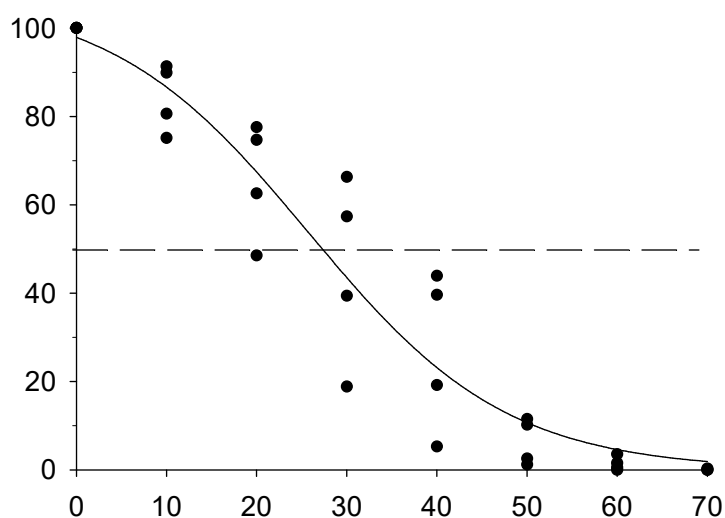

E) *M. trossulus*

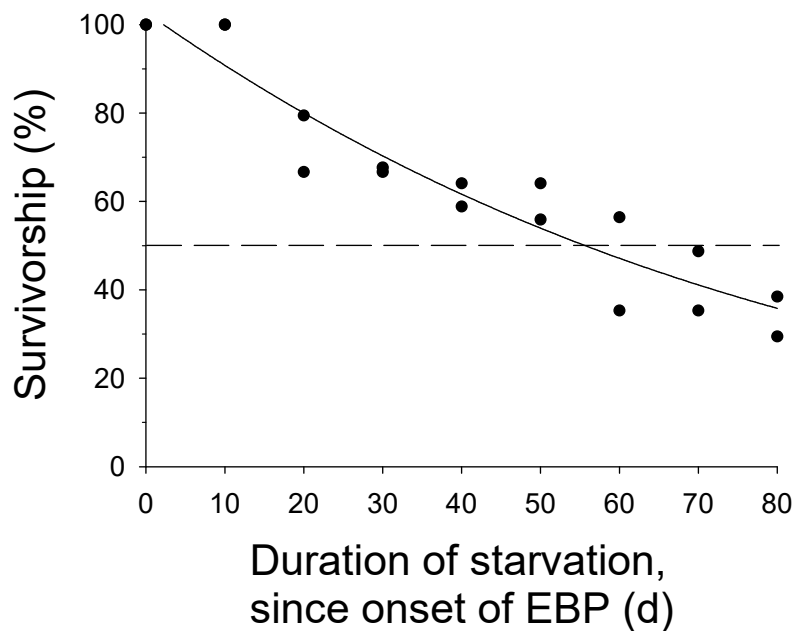

F) *Petrolisthes* spp.

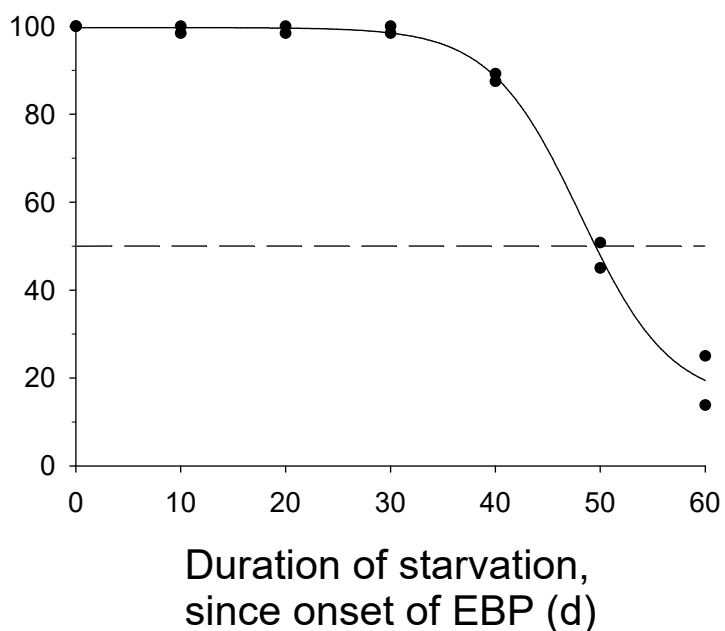

Supplement: Supplementary file 1 — Fig S1 [file ECE3-11-8882-s002.pdf]
