# Supplementary material for: Role of depleted initial energy reserves in early benthic phase mortality of six marine invertebrate species
Source: Ecol Evol. 2021 Jun 6;11(13):8882–96. doi: 10.1002/ece3.7723 (PMC8258192; doi:10.1002/ece3.7723)
Supplement: Supplementary file 2 — Appendix S1 [file ECE3-11-8882-s001.docx]

APPENDIX A.

**Table A1.** Number of batches and number of individuals in each batch for all six benthic invertebrate species in the experiment assessing the survivorship of EBP individuals unable to replenish energy reserves.

| Species | Batch # | # individuals | Total # of individuals |
| --- | --- | --- | --- |
| *B. glandula* | 1 | 16 | 249 |
|  | 2 | 41 |  |
|  | 3 | 34 |  |
|  | 4 | 9 |  |
|  | 5 | 21 |  |
|  | 6 | 88 |  |
|  | 7 | 25 |  |
|  | 8 | 15 |  |
| *C. dalli* | 1 | 20 | 269 |
|  | 2 | 34 |  |
|  | 3 | 17 |  |
|  | 4 | 27 |  |
|  | 5 | 171 |  |
| *N. ostrina* | 1 | 29 | 449 |
|  | 2 | 90 |  |
|  | 3 | 37 |  |
|  | 4 | 131 |  |
|  | 5 | 21 |  |
|  | 6 | 68 |  |
|  | 7 | 47 |  |
| *N. lamellosa* | 1 | 196 | 930 |
|  | 2 | 198 |  |
|  | 3 | 170 |  |
|  | 4 | 200 |  |
|  | 5 | 166 |  |
| *M. trossulus* | 1 | 34 | 73 |
|  | 2 | 39 |  |
| *Petrolisthes* spp. | 1 | 72 | 112 |
|  | 2 | 40 |  |

**Table A2.** GLM equations for the relationship between % survivorship and duration of starvation, starting at the onset of the EBP, for all six species.

| Species | Equation |
| --- | --- |
| *B. glandula* | y=1/(1+exp(-5.2253+0.0666x)) |
| *C. dalli* | y=1/(1+exp(-4.7558+0.0686x)) |
| *N. ostrina* | y=1/(1+exp(-3.3032+0.0591x)) |
| *N. lamellosa* | y=1/(1+exp(-3.1759+0.1139x)) |
| *M. trossulus* | y=1/(1+exp(-2.4995+0.0419x)) |
| *Petrolisthes spp.* | y=1/(1+exp(-8.7306+0.1720x)) |

**Table A3.** Starvation LD_20_ and percent survivorship at 5 d starvation, and their associated standard errors (SE), calculated from generalized linear models (GLM) of survival as a function of duration of starvation for each size class of each batch of *Nucella ostrina* hatchlings. n represents sample size for each size class of each batch.

| Batch | Size class | n | Starvation LD20 (d) | SE | Survivorship (%) at 5 d starvation | SE |
| --- | --- | --- | --- | --- | --- | --- |
| 1 | Small | 13 | 20.4 | 5.8 | 90.5 | 3.9 |
|  | Medium | 29 | 44.7 | 8.7 | 93.9 | 2.6 |
| 2 | Small | 106 | 21.6 | 1.4 | 94.8 | 1.0 |
|  | Medium | 90 | 44.3 | 1.6 | 98.9 | 0.4 |
|  | Large | 217 | 69.0 | 3.4 | 97.6 | 0.5 |
| 3 | Medium | 37 | 20.7 | 3.4 | 94.0 | 2.6 |
|  | Large | 56 | 38.3 | 3.0 | 94.6 | 1.5 |
| 4 | Large | 59 | 31.9 | 2.8 | 93.9 | 1.6 |
| 5 | Small | 11 | 26.3 | 3.9 | 97.6 | 2.1 |
|  | Medium | 25 | 22.7 | 6.7 | 87.8 | 3.9 |
|  | Large | 15 | 32.3 | 7.9 | 91.5 | 4.3 |
| 6 | Small | 57 | 12.9 | 2.2 | 88.9 | 2.5 |
|  | Medium | 152 | 26.1 | 1.6 | 93.1 | 1.2 |
|  | Large | 76 | 32.1 | 2.5 | 94.1 | 1.5 |
| 7 | Small | 34 | 10.8 | 2.5 | 87.9 | 3.4 |
|  | Medium | 115 | 44.8 | 3.5 | 95.6 | 1.1 |
|  | Large | 85 | 49.1 | 2.8 | 99.3 | 0.4 |
| 8 | Small | 68 | 30.2 | 2.1 | 96.5 | 1.3 |
|  | Large | 34 | 39.1 | 3.5 | 99.3 | 0.7 |

**Table A4.** Recovery LD_50_ and associated standard error (SE) from linear model analysis of the proportion of individuals able to recover from different durations of starvation. The z statistic represents the strength of the relationship between the % survivorship and the duration of starvation; df = degrees of freedom.

| Species | Recovery LD_50_ (d) | SE | F value | n | R^2^ | p |
| --- | --- | --- | --- | --- | --- | --- |
| *C. dalli* | 28.6 | 4.2 | 17.56 | 6 | 0.81 | 0.014 |
| *N. ostrina* | 20.9 | 6.6 | 7.08 | 6 | 0.64 | 0.056 |

|  |  |
| --- | --- |
|  |  |
|  |  |

**Figure A1.** Relationship between % survivorship and the duration of starvation, starting at the onset of the EBP, for five species of benthic invertebrates. Results from the generalized linear models (GLMs) are shown in Table 1 in Chapter 2.
